# Causes of Oral Granulomatous Disorders: An Update and Narrative Review of the Literature

**DOI:** 10.1007/s12105-024-01678-7

**Published:** 2024-08-07

**Authors:** Waleed A. Alamoudi, Rafik A. Abdelsayed, Thomas P. Sollecito, Ghaida A. Alhassan, Roopali Kulkarni, Mohammed A. Bindakhil

**Affiliations:** 1 Department of Oral Diagnostic Sciences, Division of Oral Medicine, King Abdulaziz University, Jeddah, Saudi Arabia; 2 Division of Oral Medicine, UCL Eastman Dental Institute, University College London, London, UK; 3 Division of Oral and Maxillofacial Pathology, Augusta University, Augusta, GA USA; 4 Department of Oral Medicine, University of Pennsylvania, Philadelphia, PA USA; 5 Division of Infectious Diseases, King Abdulaziz Medical City, Riyadh, Saudi Arabia; 6 Department of Oral and Maxillofacial Surgery and Diagnostic Sciences, College of Medicine and Dentistry, Riyadh Elm University, Riyadh, Saudi Arabia; 7 Division of Oral Medicine, Augusta University, Augusta, GA USA

**Keywords:** Oral granulomatous disorders, Oral granulomatous reaction, causes of oral granulomatous disease, Oral granuloma

## Abstract

Granulomatous diseases include a diverse range of chronic inflammatory disorders with a wide variety of pathologies and clinical characteristics. In particular, the orofacial region can be affected by granulomatous conditions—whether as an isolated disease or as part of a systemic disorder. Regardless of the nature of the disease or its mechanism of development, precise diagnosis can be challenging, as etiopathogenesis may be driven by several causes. These include reactions to foreign bodies, infections, immune dysregulation, proliferative disorders,, medications, illicit drugs, and hereditary disorders. Granulomas can be identified using histopathological assessment but are not pathognomonic of a specific disease, and therefore require correlation between clinical, serological, radiographical, and histopathological findings. The purpose of this review is to provide a summary of the etiopathogenesis, clinical and histopathologic characteristics, and treatment of oral granulomatous disorders.

## Introduction

Granulomatous disorders include a wide range of diseases that share the histopathological hallmark of granuloma development. A granulomatous reaction is described as an overstimulation of the immune system that results in Type IV (delayed) hypersensitivity to an array of factors: including reactions to foreign exogenous or endogenous substances, infection, immune-mediated dysregulation, drug-induced reactions, and malignancy [1–4]. The clinical manifestations of granulomatous disorders varies based on the affected organs; but the lungs, kidneys, and skin are commonly involved [1]. The orofacial region is frequently impacted as well, and foreign materials are the most prevalent causes of localized granulomatous reactions [5].

Granulomatous inflammation is a unique chronic inflammation that develops as a result of antigen resistance to inflammatory cells, or ‘first responders’—in particular, neutrophils and eosinophils [1]. A granuloma is a histologic finding defined as a cluster of inflammatory cells. It primarily consists of macrophages (epithelioid histiocytes), which usually merge to create multinucleated giant cells (Langhans giant cells) [6]. Other inflammatory cells, such as lymphocytes, neutrophils, eosinophils, and fibroblasts, may also be observed in granulomatous inflammation.

Various histopathological features of granulomas—including the presence of necrosis, intracytoplasmic calcifications with multinucleated giant cells (Schaumann bodies), and the demonstration of a vasculitic reaction—can be crucial in reaching a definitive diagnosis [1, 3].

Despite its importance, identifying the underlying etiology of a granulomatous disorder can be challenging, especially in atypical clinical presentation or cases without an obvious foreign object or identifiable infectious pathogen [2]. This review highlights the most common local and systemic factors that trigger granulomatous oral diseases and reports additional causal factors: such as proliferative disorders, medications, illicit drug use, and hereditary disorders.

## Reactions to Foreign Materials

The most common causes of orofacial granulomatous disorder are foreign bodies, which can include a variety of endogenous or exogenous substances [2, 7, 8]. Hair fibers, keratin clusters, and fatty substances are examples of endogenous substances; in contrast, cosmetic materials such as hyaluronic acid filler and dental materials like amalgam fillings, impression materials, teeth polishing materials, and sutures are examples of exogenous materials that can stimulate granuloma formation.

**Fig. 1:**
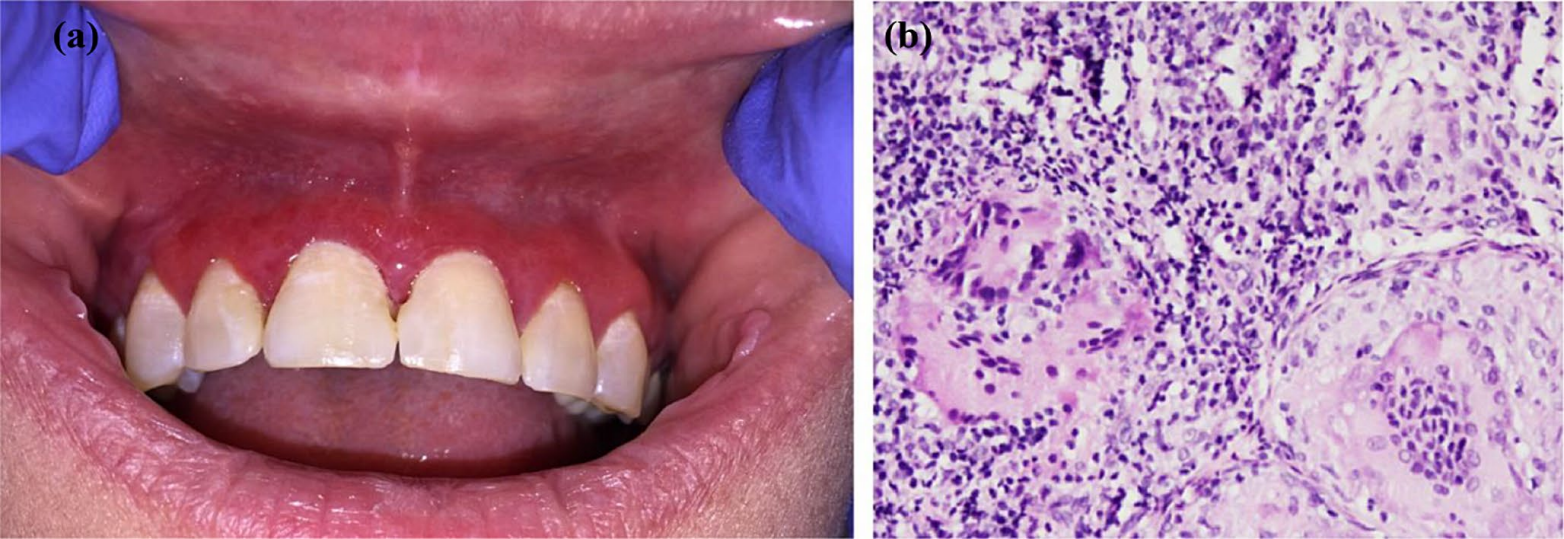
(**a**) A 46-year-old woman developed diffuse gingival swelling, erythema and bleeding a few weeks after dental hygiene visit, during which teeth polishing materials were used. (**b**) H & E section showing diffuse mononuclear cell inflammatory infiltrates interspersed by non-necrotizing granulomas

**Fig. 2:**
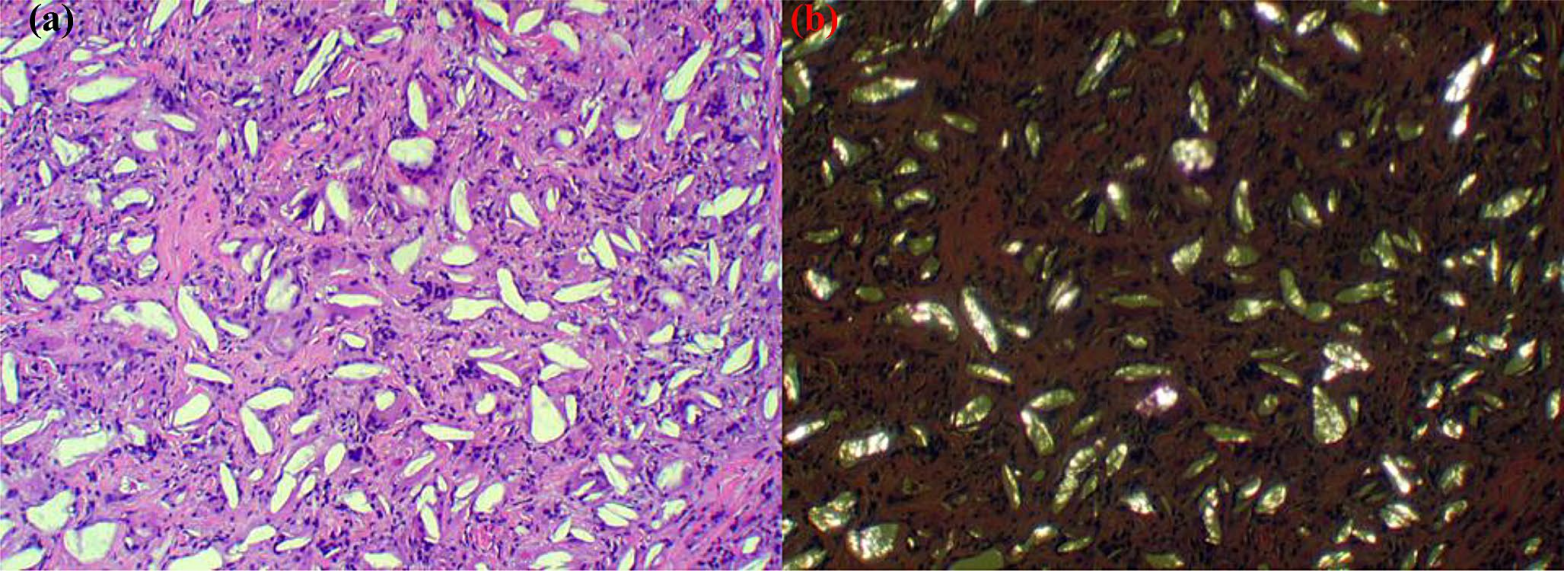
(**a**) Granulomatous reaction of epithelioid macrophages and multinucleated giant cells and spindle-shaped spaces. (**b**) Polarized light microscopy shows spindle-shaped birefringent spaces consistent with cosmetic injectable material (Sculptra)

The clinical manifestations of foreign bodies in the oral cavity are largely non-specific. They include swelling, erythema, or ulcerations of the lips, gingiva, or mucosal areas; and such swelling can be diffused or limited to the site of the foreign body (Fig. 1a) [5, 9]. A biopsy is essential in making a diagnosis, and evidence of foreign substances in the sample should confirm the diagnosis (Fig. 2a and b) [2]. Additionally, microscopic examination typically unveils the presence of unorganized basophilic material encircled by macrophages and multi-nucleated giant cells (Fig. 1b) [2, 9]. Nevertheless, failure to microscopically detect foreign bodies does not rule them out as a source of the disease, as not all foreign materials are polarizable.

The signs and symptoms typically resolve following surgical excision of the foreign material. The administration of topical or intralesional corticosteroids have also been utilized in treatment [9]. Further evaluation is necessary to rule out other causes of granulomatous inflammation if the signs or symptoms persist.

## Infection

### Tuberculosis

Tuberculosis (TB) is a chronic bacterial infectious disease induced by airborne transmission of *Mycobacterium tuberculosis* (MBT) [10]. Epidemiological studies indicate that the percentage of new TB cases might be declining, but the rise of multidrug-resistant tuberculosis and total drug-resistant tuberculosis poses a danger to disease management [11].

The clinical presentation of TB varies, but is usually limited to respiratory symptoms. This is referred to as pulmonary TB, whereas miliary or disseminated TB are identified when other parts of the body are involved. Oral involvement of TB is related to extrapulmonary or disseminated variants, which comprise 0.05–5% of all cases [11]. Multiple sites in the oral cavity can be affected by TB, with the buccal mucosa, hard and soft palate, gingiva, lips, and tongue comprising the most frequently affected sites [12].

The most common oral presentation is isolated ulceration with poorly-defined margins [12]. Other reported manifestations include gingival hyperplasia, jaw osteomyelitis, and nodules. A biopsy from oral lesions typically reveals a necrotizing granulomatous reaction and MBT organisms. However, necrotizing granulomatous inflammation may not occur in early lesions or in immunocompromised patients [13]. Additional diagnostic methods—such as tuberculin skin testing, QuantiFERON gold, sputum culture collection, PCR, and chest imaging—can be used to confirm a diagnosis of TB [14]. While MBT is most commonly linked to TB, other mycobacterial pathogens have been associated with orofacial granulomatous diseases, such as *Mycobacterium leprae*, *Mycobacterium bovis*, *Mycobacterium avium complex*, and *Mycobacterium marinum* [1, 15]. Management of oral lesions associated with TB is often challenging, especially with the rise of drug-resistant strains. Collaboration with infectious disease specialists is necessary and typically requires treatment with rifampicin, isoniazid, ethambutol, and pyrazinamide (for 6–9 months) [10].

### Syphilis

Syphilis is a chronic, sexually transmitted bacterial infection induced by *Treponema pallidum (TP)* [16]. Acquired syphilis can be divided into four developmental stages: primary, secondary, latent, and tertiary. An estimated five million new syphilis cases are detected worldwide every year [17].

Primary syphilis pertains to the manifestation of an initial or primary lesion at the location of infection inoculation [17]. The main clinical sign of primary syphilis is the appearance of a painless, generally isolated sore, also called a chancre sore, which arises about 2–3 weeks after direct contact with TP. The oral cavity is infrequently affected by primary syphilis; but when it occurs, a solitary ulcer can be observed, typically on the lip and less commonly on the tongue [18, 19]. Primary lesion normally lasts between three and six weeks and can heal spontaneously.

The most frequently-observed clinical type of syphilis is secondary syphilis [17]. The typical presentation of secondary syphilis involves the appearance of non-painful flat rashes that exhibit a remarkable degree of variability in size and presentation, with the potential to occur in either a localized or diffuse manner. These affect both cutaneous and mucosal surfaces [17]. Oral lesions are observed in a minimum of 30% of individuals diagnosed with secondary syphilis [18, 19]. Mucous patches and maculopapular lesions are the two most common oral manifestations of secondary syphilis, although nodular lesions can occur in rare cases [18]. The mucous patches typically present as erosions or ulcers with an oval-to-crescent shape that measure approximately 1 cm in diameter. Snail-track-like ulcers can appear when the mucous patches fuse together.

The symptoms at this stage can also resolve spontaneously without active treatment, although complete resolution requires weeks to months. Following the resolution of the secondary disease, untreated syphilis progresses to a latent phase characterized by an absence of clinical manifestations [17]. Approximately one-third of individuals who have not received treatment during the latent phase will develop tertiary syphilis, which exhibits features such as late neurosyphilis and cardiovascular involvement [17].

**Fig. 3:**
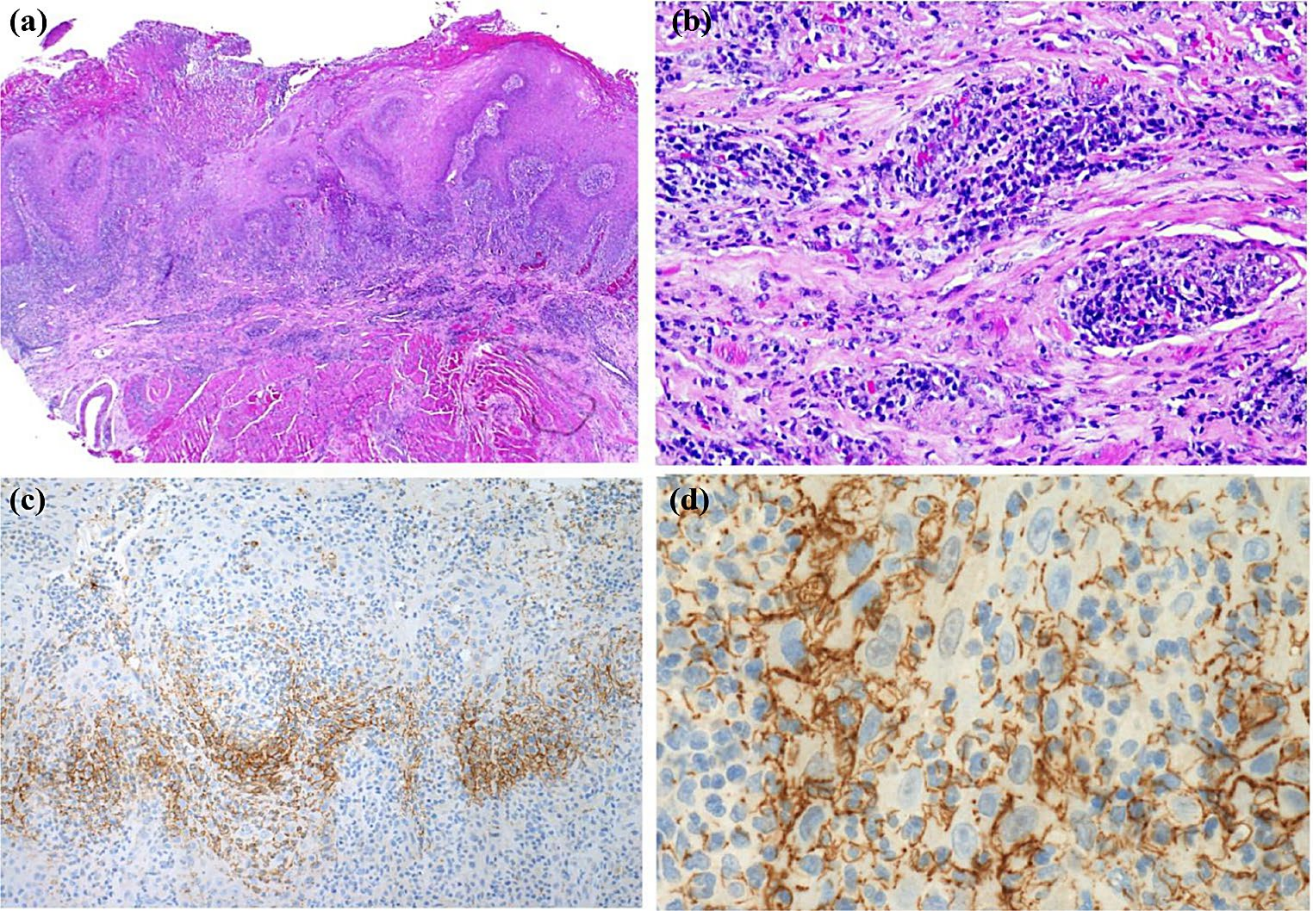
(**a**) Tongue biopsy showing ulceration with diffuse intraepithelial neutrophilic exocytosis and stromal diffuse inflammation (low power H&E). (**b**) Perivascular plasmacytic infiltrates (high power H&E). 9**c** and **d**) Sections stained with immunoperoxidase stain showing numerous spirochetes of Treponema pallidum)

Since the clinical signs and symptoms of syphilis may be inconspicuous and mimic several other diseases, syphilis is known as the ‘great imitator.’ Serologic testing, such as non-treponemal tests, e.g. rapid plasma reagin (RPR) and venereal disease research laboratory (VDRL), and treponemal immunoassay tests can be used for the diagnosis of syphilis [20]. The histopathologic characteristics of oral syphilitic lesions vary, but typically include abnormal perivascular inflammation with a predominance of plasma cells, regardless of the presence or absence of granulomatous inflammation, which typically can be seen in tertiary syphilis. Furthermore, immunohistochemistry stains can aid in the detection of spirochetes (Fig. 3) [21]. The therapy of choice for all phases of syphilis is benzathine penicillin G [20].

### Histoplasmosis

Histoplasmosis is an infectious disease caused by *Histoplasma capsulatum* (HC), a saprophytic and dimorphic fungus [22]. HC is predominantly found in areas where soil has been exposed to bird or bat feces, especially when such soil is unsettled due to either man-made activities or natural occurrences [22].

Most people exposed to HC are either asymptomatic or only experience a very mild sickness. Histoplasmosis is classified into three variants: acute, chronic, and disseminated [23]. The acute form usually presents as a mild lung infection that resolves spontaneously, while the chronic type resembles TB in its symptoms and predominantly affects the lungs [23]. In contrast, a disseminated infection can spread to numerous parts of the body. Disseminated forms usually appear in elderly and immunocompromised individuals and can cause serious complications.

**Fig. 4:**
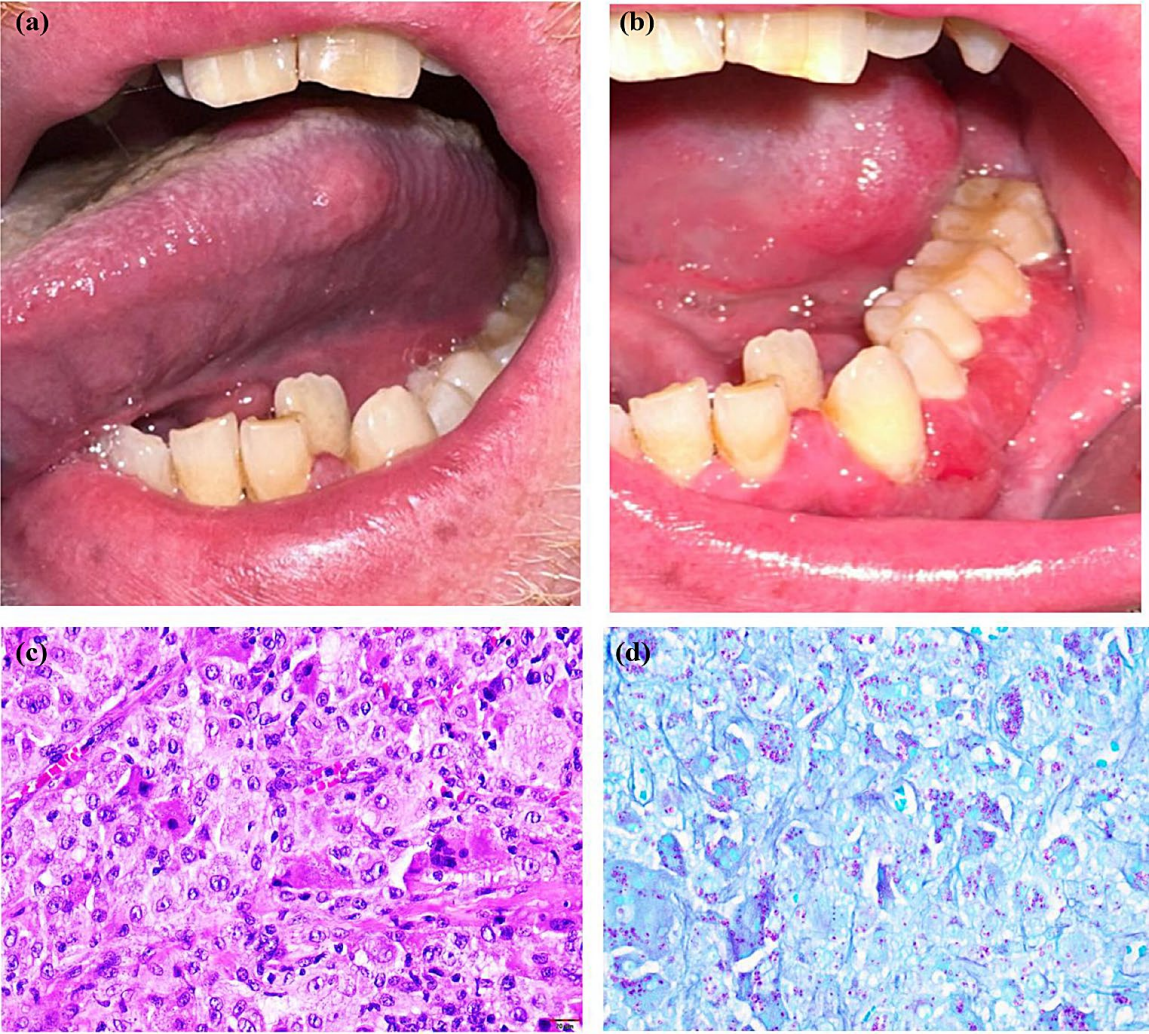
(**a**) Nodule on the lateral border on the tongue in a patient with disseminated histoplasmosis. (**b**) Gingival involvement of histoplasmosis causing swelling, erythema, and ulceration. (**c**) Histologic sections showing densely-packed sheet of mononuclear and multinucleated giant cells with intracytoplasmic organisms. (**d**) PAS-stained sections showing mononuclear and multinucleated giant cells with intracytoplasmic clusters of fungus morphologically consistent with *Histoplamsa capsulatum*

**Fig. 5:**
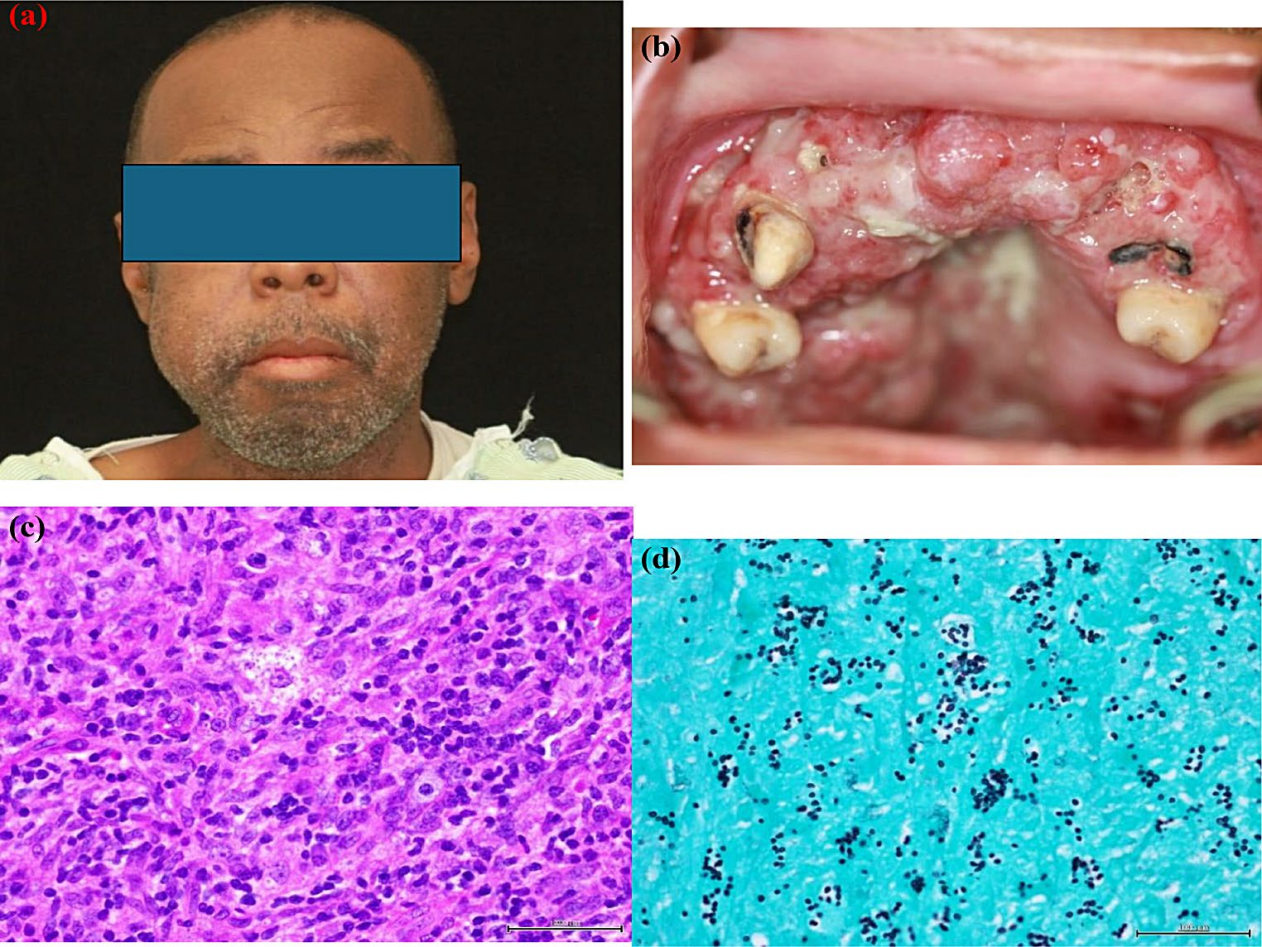
(**a**) 46-year-old male with history of HIV/AIDS presented with facial swelling admitted to hospital, then referred to oral maxillofacial clinic for diffuse lesions in the oral cavity. (**b**) Widespread raised and rough/soft lesions involving the upper alveolar gingiva and soft palate. Red and white febrile with spontaneous bleeding. (**c**) Histologic sections showing densely-packed sheet of macrophages and multinucleated giant cells with intracytoplasmic *Histoplasma capsulatum*. (**d**) Sections showing mononuclear and multinucleated giant cells with intracytoplasmic clusters of fungus morphologically consistent with *Histoplamsa capsulatum*

Oral mucosal lesions can be seen in the disseminated form and may be localized or widespread [24, 25]. The oral manifestations have the potential to appear on any region of the oral mucosa, although they are most frequently observed on the palate, buccal mucosa, gingiva and tongue (Fig. 4a and b) (Fig. 5a and b) [23]. The most common type of oral lesion observed in histoplasmosis is a persistent ulceration that may resemble oral cancer, necessitating a biopsy to rule out malignancy.

Various diagnostic methods are available to confirm the diagnosis of histoplasmosis, including serology tests, tissue cultures, PCR, and biopsy [24]. The most reliable method for diagnosing histoplasmosis is tissue biopsy [24]. Upon microscopic examination of oral lesions, histoplasmosis generally reveals infiltration of lymphocytes and histiocytes, as well as fungal components that are roughly 2–4 μm in size. These components, encircled by a notable translucent halo, can be identified within macrophages, multinucleated giant cells, or dense connective tissue (Fig. 4c) (Fig. 5c) [23, 26]. Although histiocytic infiltration is typical, the formation of distinct granulomas is an uncommon occurrence [23]. To determine the presence of fungus in the tissues, the use of specific staining methods like Periodic acid-Schiff (PAS) and Grocott’s methenamine silver (GMS) become necessary (Fig. 4d) [26]. Amphotericin B combined with itraconazole is commonly prescribed to treat severe and disseminated infections, while itraconazole alone can be administered to treat mild to moderate cases [27].

### Other Fungal Infections

Recent research has indicated a rise in the prevalence of fungal infections, such as mucormycosis, involving the maxillofacial region among immunocompromised patients at the time of the coronavirus disease 2019 (COVID-19) pandemic [28]. The risk is higher in hospitalized patients with diabetes who are treated with corticosteroids for COVID-19 related acute respiratory distress syndrome. The immunosuppressive properties of corticosteroids—combined with the presence of microangiopathy in individuals with diabetes and potential peripheral microthrombi in COVID-19 patients—creates an environment favorable to the development of mucormycosis. Typically, clinical manifestations include visual impairment, sinus inflammation, increased intracranial pressure, and maxillary ulceration and necrosis. The conclusive identification of mucormycosis requires a biopsy specimen, while culture examination serves as a supplementary method [28].

Several other fungal organisms can lead to inflammation with granulomatous changes of the oral mucosa, such as *Blastomyces dermatitidis*, *Cryptococcus neoformans*,* Coccidioides immitis*, and *Rhinosporidium seeberi*, all of which may result in comparable clinical manifestations [2, 29]. Recent reports have also indicated that *Paracoccidioides brasiliensis* and *Mucor* may be associated with the existence of multinucleated giant cells and the development of granulomatous inflammation [30, 31]. Although the diagnosis of invasive fungal infections can be achieved utilizing histopathologic evaluation, correlation with fungal microbiology such as cultures and antigens tests is recommended. This is particularly important in the absence of evidence of fungal microorganisms in histopathology, with persistent clinical suspicion of fungal infection.

### Immune-Mediated Causes

#### Sarcoidosis

Sarcoidosis is a multi-organ inflammatory disorder that creates non-necrotizing granulomas in various parts of the body, with the lungs being the most commonly impacted organs [32]. The yearly incidence of sarcoidosis demonstrates regional variability, ranging from 1 to 15 cases per 100,000 individuals. The prevalence rates exhibit the lowest values in Eastern Asian countries, with greater values in North America and Australia, and the highest prevalence in Scandinavian countries [33]. Black individuals and women seem to be more frequently affected by sarcoidosis. The exact etiology of sarcoidosis is poorly characterized. Some individuals appear to have a hereditary susceptibility to the condition, having an immune dysregulation reaction stimulated by either a commensal respiratory pathogen-related antigen or environmental factors [32].

Sarcoidosis has a wide range of clinical manifestations [34]. The lungs are often the most prominent organs to be affected, and clinical manifestations may include a chronic dry cough, dyspnea, and chest pain [34]. Approximately one-third of patients with sarcoidosis develop cutaneous lesions, such as erythema nodosum and maculopapular nodules or plaques [34]. Lupus pernio is a skin condition associated with sarcoidosis, which manifests as reddish-blue plaques and nodules on the head and neck region, most notably on the cheeks, nose, and ears. Other disease features include ocular sarcoidosis, cardiac disease, joint pain, and hepatosplenomegaly [34, 35].

**Fig. 6:**
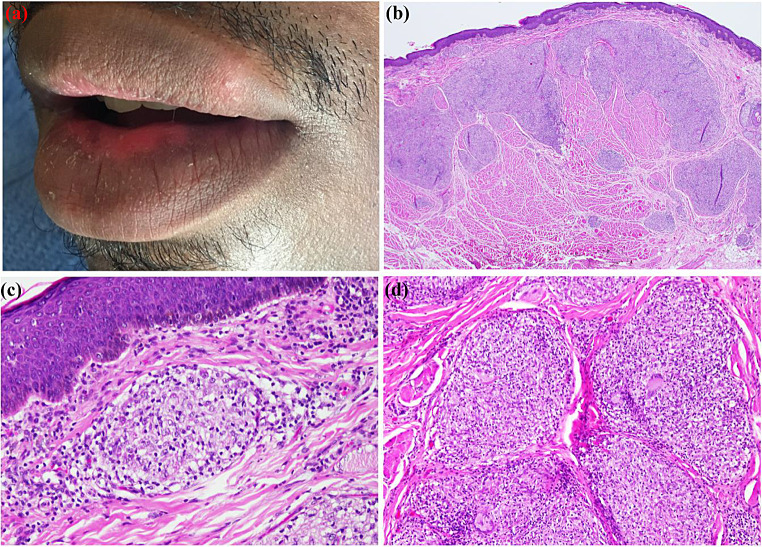
(**a**) A 30-year-old man with tender facial plaques and papules on upper lip and skin. **b**, (**c**) and (**d**) Upper lip biopsy showing non-necrotizing granulomas. The patient was later diagnosed with sarcoidosis

**Fig. 7:**
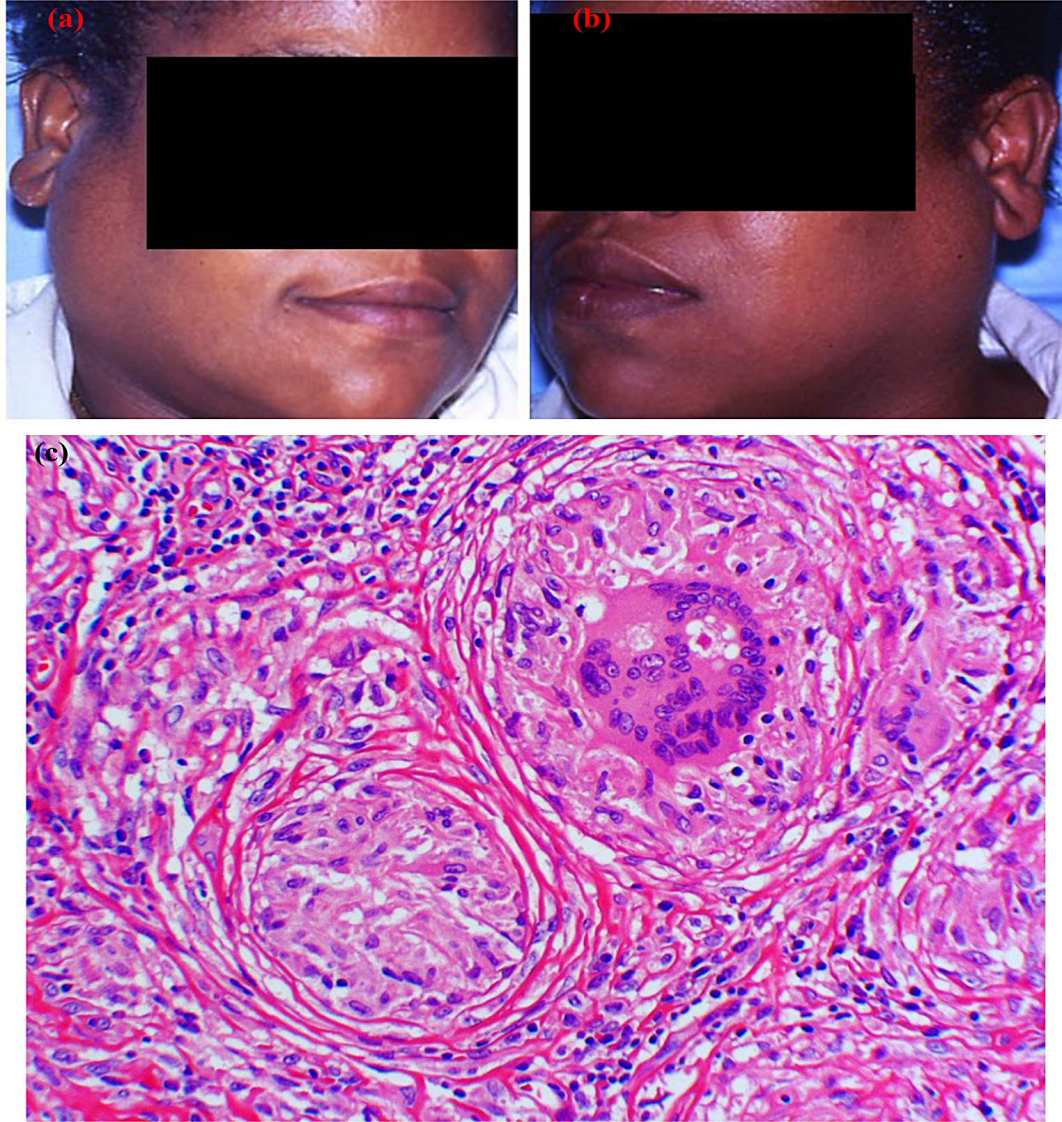
(**a**) A 38-year-old female with s and bilateral parotid enlargement later diagnosed with sarcoidosis. (**b**) Core biopsy of the parotid showing non-necrotizing granulomas. Multinucleated giant cells within granuloma with intracytoplasmic asteroid body (stellate crystalline inclusions) are noted

Although oral involvement of sarcoidosis is uncommon, oral lesions can arise as the first sign of sarcoidosis, sometimes years before systemic development; otherwise, they may simultaneously occur with other systemic manifestations [36, 37]. Sarcoidosis can affect different parts of the mouth and give rise to alveolar bone necrosis, tooth loss, mucosal ulcers, gingival swelling, and lip plaques or papules (Fig. 6) [36]. Salivary gland involvement has been correlated with sarcoidosis, presenting as parotid enlargement associated with dry mouth (Fig. 7a). Heerfordt syndrome is a rare manifestation of glandular involvement and is considered pathognomonic for sarcoidosis. This syndrome is characterized by systemic sarcoidosis associated with (typically bilateral) parotitis, facial palsy, and uveitis [36].

The diagnosis of sarcoidosis can be established based on several factors: the presence of clinical and radiographic abnormalities, the histopathological presence of non-necrotizing granuloma, and the exclusion of other potential systemic etiologies with similar clinical features [35]. An oral or salivary gland biopsy can provide valuable insights, as it can reveal the presence of granulomas consisting of epithelioid histiocyte aggregations which may form multinucleated giant cells (Fig. 7b) [37]. Angiotensin-converting enzyme (ACE) levels may be raised in sarcoidosis, but this increase is neither specific nor sensitive [35]. Additionally, hilar lymphadenopathy on chest x-rays can be suggestive of sarcoidosis. Sarcoidosis is typically treated with corticosteroids and immunosuppressive medications; oral disease is managed with topical and intralesional corticosteroids in mild to moderate cases, and systemic immunosuppressants are considered for severe oral involvement [35, 36].

### Crohn’s Disease

Crohn’s disease (CD) is a chronic inflammatory bowel disease of unclear etiology [38]. CD is estimated to affect around 200 out of every 100,000 individuals, with a yearly incidence of 3 to 20 cases per 100,000 individuals [39]. Although CD may appear at any age, patients typically exhibit the signs and symptoms as teenagers or in early adulthood. Males and females seem to be equally affected, but males are more commonly involved at a younger age [38, 39]. The disease is widespread across North America and Western Europe, but the frequency appears to be rising in the developing world as well [39]. The etiopathogenesis of CD has been inadequately elucidated; however, numerous environmental, immunological, and microbiological factors may play a role in its pathogenesis or exacerbation among genetically predisposed persons [38, 39].

**Fig. 8:**
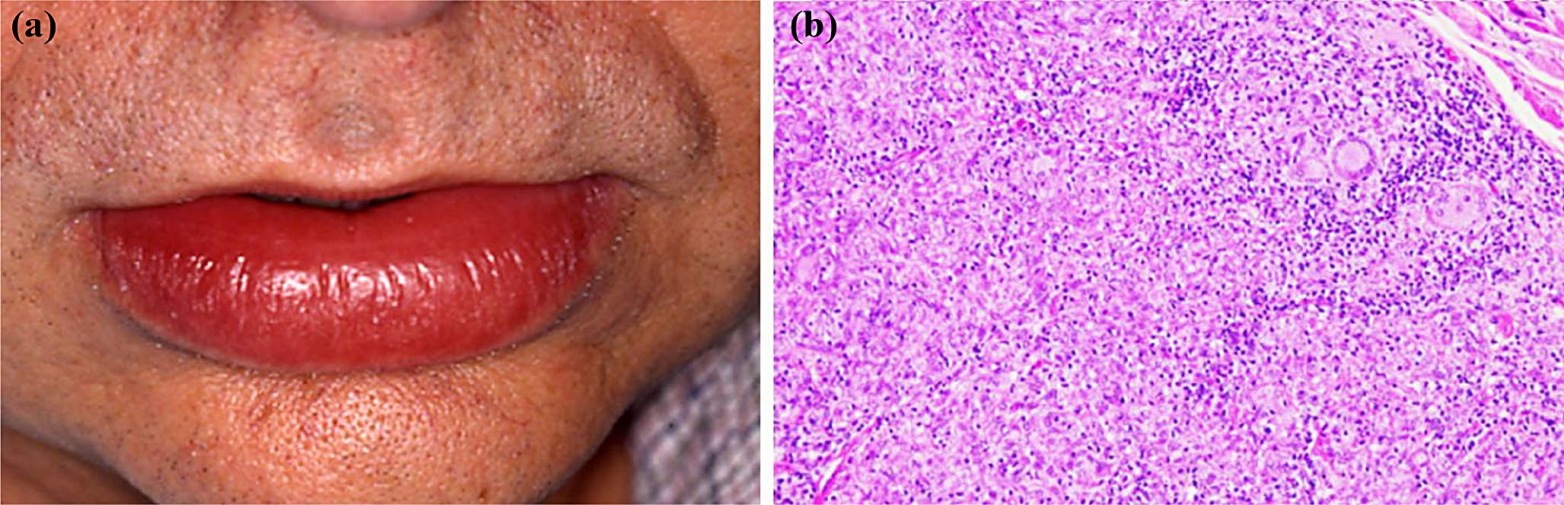
(**a**) A 52-year-old man with persistent lower lip swelling for more than 2 months. Medical history at presentation was non-contributory. He complained of abdominal cramps and diarrhea. (**b**) Lip biopsy showing non-necrotizing granulomas, composed of clusters of epithelioid histiocytes and multinucleated giant cells. The patient was later diagnosed with Crohn’s disease after gastroenterology workup

**Fig. 9:**
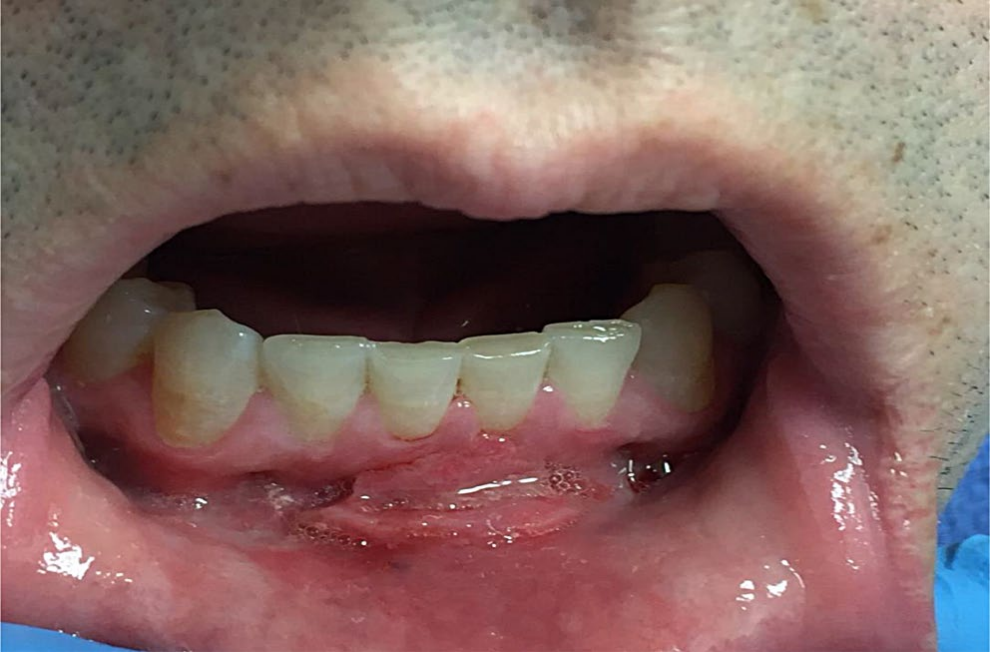
Deep linear ulceration with raised and hyperplastic borders in a patient with Crohn’s disease

CD exhibits diverse clinical presentations, and patients often manifest non-specific signs and symptoms—such as abdominal pain, cramping, diarrhea, malnutrition, arthralgia, and weight loss [39]. It is estimated that nearly one-third of CD patients develop at least one extraintestinal disease, such as oral lesions, at some point of the disease’s course [40]. Oral lesions may be the presenting sign, preceding GI disease in approximately 60% of cases (Fig. 8) [41]. CD can arise in various parts of the oral cavity, involving the lips, buccal mucosa, gingiva, and retromolar and vestibular areas [40]. The oral manifestations can be categorized as either specific or non-specific, with the latter being more prevalent. Disease-specific lesions include deep linear ulceration, lip swelling with vertical fissures, gingival swelling, buccal and labial mucosa swelling (with a cobblestone-like presentation), and indurated tag-like lesions (Fig. 9) [40]. Non-specific oral lesions include aphthous-like stomatitis and pyostomatitis vegetans [40].

A CD diagnosis is established via clinical, endoscopic, pathologic, radiographic, and laboratory studies in a multidisciplinary approach [38]. Examining oral biopsy samples can reveal the presence of chronic inflammatory cell infiltration as well as non-necrotizing granulomas, primarily consisting of epithelioid histiocytes and lymphocytes [42]. A colonoscopy may reveal evidence of skip lesions and the remarkable presence of transmural inflammation histologically [38]. Serological assessment of certain biomarkers, such as anti-*Saccharomyces cerevisiae* antibodies (ASCA), is advisable [43]. Additionally, antineutrophil cytoplasmic antibody (ANCA) may be detected in CD cases, although it is more commonly associated with ulcerative colitis [43]. Several treatments involving immunosuppressants and immunotherapy, such as infliximab, adalimumab and ustekinumab, have been used to treat patients with CD [44]. Topical corticosteroids are the treatment of choice for mild oral ulcerations, whereas extensive oral lesions can be treated with intralesional corticosteroid injections, systemic corticosteroids, and immunosuppressants [40].

### Orofacial Granulomatosis

Orofacial granulomatosis (OFG) is a rare disease characterized by chronic swelling of the lips and facial areas, as well as swelling and ulceration in oral mucosal tissues [45]. This condition usually occurs in children and young adults, with no gender or racial inclination [45]. Research indicates that a subset of individuals with OFG, particularly those who experience the disease’s onset in childhood, may ultimately develop CD or, less frequently, sarcoidosis [45]. The precise etiology of OFG is still unidentified [46]. However, in combination with genetic predisposition, hypersensitivity to certain factors—such as dental hygiene products and materials, infections, and certain dietary ingredients—have been suggested as a possible mechanism [46].

**Fig. 10:**
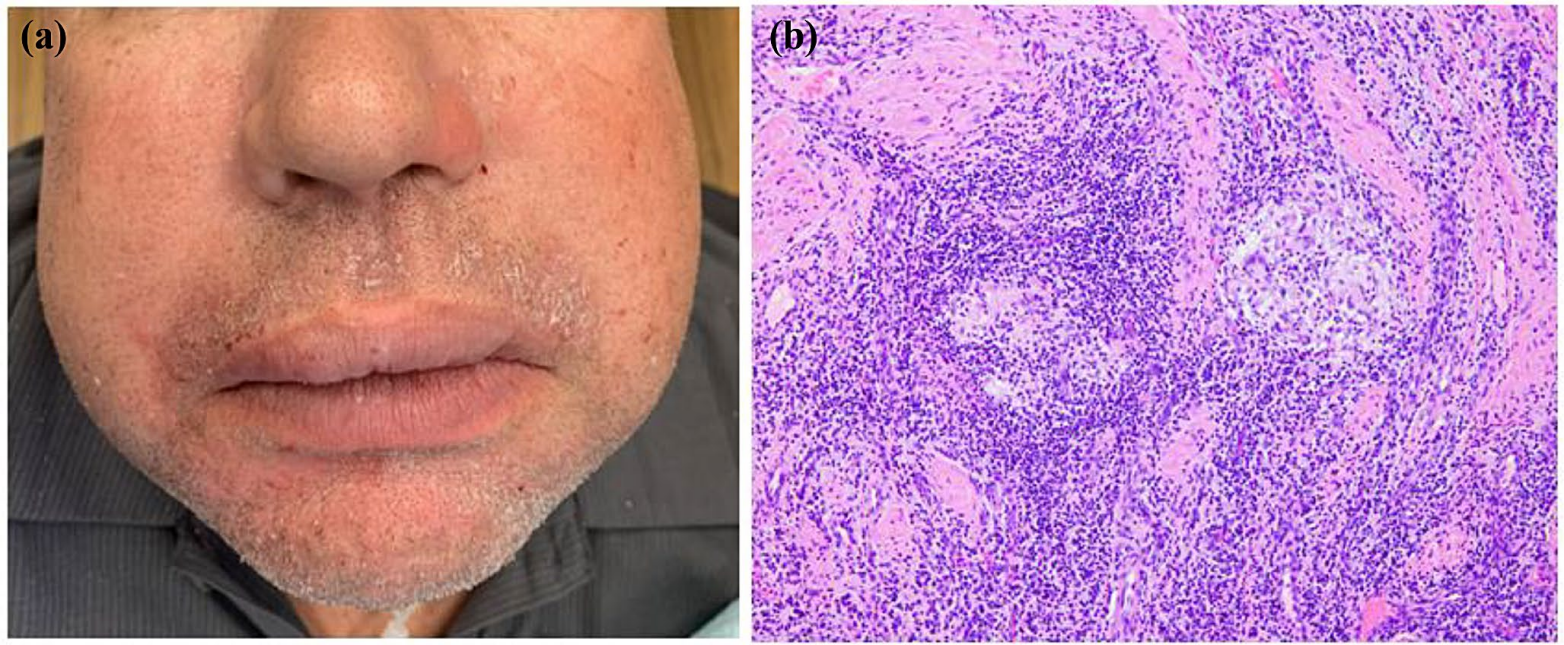
(**a**) A 48-year-old man presented with progressive diffuse facial and lip swelling present for several weeks. (**b**) Oral biopsy showing numerous non-necrotizing granulomas with hyperplastic fibrous stroma (H&E stained)

Clinically, the most prominent feature of OFG is labial enlargement, which is observed in over 90% of patients [45]. Facial swelling has been found among patients with OFG affecting various sites, such as the cheeks, forehead, and eyelids [45]. Several intraoral manifestations of OFG have been reported, including diffuse swelling of oral mucosa, mucosal tags, ulceration, and a fissured tongue, with fissures that gives rise to a cobblestone-like presentation (Fig. 10a) [45]. It has been recorded that a certain subset of individuals diagnosed with OFG may experience facial palsy, which typically occurs unilaterally and can be associated with otalgia and altered taste [45]. The presence of lip swelling and a fissured tongue in conjunction with facial palsy is clinically described as Melkersson–Rosenthal syndrome, which is classified as a subtype of OFG [45].

A definitive diagnosis of OFG is made when the clinical orofacial characteristics are noted, and a possible local or systemic granulomatous disorders are precluded [47]. Although the demonstration of a non-necrotizing granuloma can be diagnostically beneficial, this histopathological finding is not essential for OFG diagnosis (Fig. 10b) [45]. Intraoral lesions can be treated with topical corticosteroids, while intralesional corticosteroid injections are typically the therapy of choice to treat labial and facial swelling [45]. Surgical intervention may be required if local treatment fails to reduce the swelling. Systemic drugs, such as corticosteroids and immunosuppressant, have been also employed to manage OFG [45, 47].

### Lichenoid Granulomatous Reaction

Lichenoid granulomatous reactions (LGR) is an exceedingly rare condition, characterized by the evidence of both granulomatous inflammation, along with a band-like lymphocytic lichenoid infiltration [48, 49]. It can appear in skin (lichenoid granulomatous dermatitis) and oral mucosa (lichenoid granulomatous stomatitis). The etiology of this condition is not fully understood, however, LGR has been observed in association with causative factors such as medications and foreign substances [48, 50]. In the mouth, the gingiva, buccal mucosa, and vestibules are the most commonly affected areas. Histopathologically, the most notable characteristic was the existence of either large collections of histiocytes or well-defined granulomas, in combination with a band-like infiltration of lymphocytes [49, 50]. While there is limited information on LGS treatment in the literature, it seems to follow similar treatment approaches and outcomes observed in oral lichen planus [50].

### Vasculitis

#### Granulomatosis with Polyangiitis

Granulomatosis with polyangiitis (GPA), formerly known as Wegener’s granulomatosis, is an autoimmune condition of small blood vessels that can affect multiple regions of the body, including the head and neck [51]. GPA’s prevalence ranges from 30 to 200 individuals per million, and the sexes seem to be affected equally [52]. GPA is usually seen among Caucasians, and patients are commonly diagnosed with the disease at an old age—frequently between 60 and 70 years of age [52]. The etiopathogenesis of GPA is characterized by the development of pathologic antineutrophil cytoplasmic antibodies (c-ANCA) as a result of immune dysregulation from disparate triggers, such as environmental factors, infections, allergies, and drugs [53]. GPA is c-ANCA related, i.e., proteinase 3, as opposed to myeloperoxidase which is perinuclear ANCA (p-ANCA) related. This combination results in robust neutrophil activation and autoimmune reaction alongside blood vessels, leading to inflammation, necrosis, and the formation of granulomas [51, 53].

Clinically, head and neck manifestations are found in approximately 90% of GPA cases and include destructive sinusitis, crusting rhinitis, nasal cartilage damage, otitis media, or scleritis [54]. Other notable clinical features encompass respiratory and renal involvement [55, 56]. Oral lesions may occur in about 10% of patients, and could be the presenting sign of undiagnosed GPA in approximately 2% of cases [57]. The oral disease can present as mucosal ulceration, palatal perforation, alveolar bone necrosis, tooth loss, oro-antral fistulas, or delayed wound healing [57]. The gingival tissues may also be involved in GPA, exhibiting erythematous granular enlargement (also described as strawberry-like gingivitis), which could represent a hallmark of GPA [57]. Salivary gland enlargement and facial paralysis have also been reported [57].

GPA can be diagnosed by identifying the relevant clinical characteristics, conducting serological assessments or radiographical studies, and examining tissue samples [58]. Blood testing to detect autoantibodies, particularly c-ANCA, is also useful for diagnosis [58]. Histologically, it is rare to observe the classical triad of vasculitis, necrosis, and granulomatous inflammation in oral biopsies. Most head and neck biopsy specimens present non-specific findings, as opposed to pulmonary biopsies that display the classical disease features. [57]. Therefore, the correlation between clinical, serological, and radiological findings is critical. Immunosuppressive medications, including glucocorticoids, cyclophosphamide, and rituximab, are frequently used in patients with GPA [59].

### Proliferative Disorders

#### Langerhans Cell Histiocytosis

Langerhans cell histiocytosis (LCH) is a rare clonal neoplastic proliferation of myeloid dendritic cells expressing a Langerhans cell (LC) phenotype [60]. The overgrowth of these cells can cause inflammation and damage to the affected organs, leading to a wide range of symptoms depending on the location and severity of the disease. The disease was formerly called Histiocytosis X, which is often used as an umbrella term to refer to a group of disorders that includes eosinophilic granuloma, chronic disseminated Langerhans disease (Hand-Schüller-Christian disease), and acute disseminated Langerhans disease (Abt-Letterer-Siwe disease) [61]. The incidence of LCH is estimated to be around 1–2 cases per million people per year [62]. While LCH can arise at any age, it is higher in children, with most cases occurring in children under the age of 10 and especially among male infants.

LCH can present as a single-system or multi-system disease. Some common clinical characteristics include skin lesions, bone pain or bone lesions, swelling of the lymph nodes, and recurrent infections [61]. Although oral involvement is unusual, it may be the only manifestation of a limited disease, an early manifestation of a clinically undetected disease, or an expression of a multiorgan disorder [63–65]. Oral lesions can display as non-specific ulceration, bone lesions, tooth loss, swelling or inflammation of the gums, and pain or numbness in the jaw or face.

The diagnosis of LCH involves the use of tools such as imaging studies, blood tests, and tissue biopsies [66]. The presence of a punched-out appearance on a bone radiograph indicates a potential association with LCH. Biopsy specimens are subjected to microscopic examination to identify an excessive quantity of Langerhans cells, which are moderately large and oval-shaped, lacking a dendritic form. They have nuclei that are grooved like coffee beans or exhibit a complex, folded appearance. Analysis of oral mucosal biopsies can reveal abnormal Langerhans cells characterized by the presence of Birbeck granules on electron microscopy, as well as an inflammatory composition that includes neutrophils, eosinophils, lymphocytes, and mononuclear histiocytes-like Langerhans cells [63–65]. Granulomas are not typically present in histopathology; however, in some cases, LCH can demonstrate granulomatous inflammation, potentially presenting as other relevant granulomatous disorders. Immunohistochemistry stains using antibodies for CD1a, S100, Langerin and Fascin may be used to aid in the diagnosis of LCH. Surgical curettage is commonly documented as the preferred elective treatment for LCH solitary bone lesions in the jaw. Alternative treatment options, such as radiotherapy, chemotherapy, and corticosteroids, have also been employed [63].

### Hereditary Causes

#### Chronic Granulomatous Disease

Chronic granulomatous disease (CGD) is a rare genetic disorder characterized by dysfunction in the immune system’s ability to fight off certain types of bacterial and fungal infections [67]. The condition is distinguished by the impaired functionality of a particular subset of white blood cells, namely phagocytes, which play a crucial role in fighting bacterial and fungal infections. As a result, people with CGD are susceptible to recurrent and severe infections [67]. The incidence of CGD is estimated to be between 1 in 200,000 to 1 in 250,000 live births worldwide [68]. Most individuals are diagnosed with CGD during their childhood years, although there are cases where the diagnosis may not occur until adulthood. The most common form of CGD is X-linked, which primarily affects males. The autosomal recessive form is the second most common type, followed by autosomal dominant CGD [67].

The clinical features of CGD vary depending on the disease subtype, but are generally indicated by recurrent infections, inflammation, and granuloma formation [67]. Respiratory tract infections, such as pneumonia, are also frequently seen. Additionally, infections affecting various organs—such as the skin, liver, gastrointestinal tract, central nervous system, and ocular structures—can be encountered. Oral lesions have been reported in patients with CGD due to their impaired immune systems and increased susceptibility to bacterial and fungal infections [69, 70]. Some of the oral lesions reported in these patients include rampant caries, recurrent abscesses, non-specific ulceration, periodontitis, and a fissured tongue [69, 70].

Making a CGD diagnosis requires a comprehensive assessment of the patient’s medical and family history, as well as a thorough physical examination [67]. An oral biopsy can reveal cellular infiltration characterized by a significant presence of plasma cells, eosinophils, and histiocytes, indicating an intense inflammatory response; granulomas and multinucleated giant cells have also been observed [69]. Treatment options include infection management, interferon-gamma use, and bone marrow transplantation [67].

### Medications

Several medications have been linked to the emergence of drug-induced granulomatous tissue reactions, which exhibit clinical and microscopic similarities to sarcoidosis [71]. Such a reaction is characterized by a systemic response that occurs simultaneously with starting the causative drug. Immune checkpoint inhibitors, highly-active antiretroviral therapy interferons, and tumor necrosis factor (TNF-α) antagonists are the pharmacological classes most frequently linked to these drug-induced sarcoidosis-like reactions (DISRs) [72]. This disorder is nearly indistinguishable from sarcoidosis, given the histopathological presence of granulomas and similar clinical presentation.

DISRs typically involve the lungs, skin, lymph nodes, and oral cavity [71]. Multiple asymptomatic, round, reddish ulcerated nodules on the hard palate were noted in a 63-year-old woman with a history of rheumatoid arthritis that had been managed with a TNF-antagonist (adalimumab), methotrexate, and folic acid [71]. Histopathologic analysis of the biopsy sample revealed the presence of pseudoepitheliomatous hyperplasia of the epithelium, accompanied by a granulomatous inflammatory process in the connective tissue. The latter was characterized by the occurrence of multiple granulomas featuring multinucleated giant cells and epithelioid macrophages, which were encircled by lymphocytes. Therefore, a diagnosis of adalimumab-induced sarcoidosis-like lesions was made. Complete resolution of the oral lesions was achieved after the discontinuation of adalimumab [71].

In another case study, a 66-year-old man was treated with adalimumab for psoriatic arthritis and presented with dysphagia, hoarseness, and tongue ulcer [73]. After ruling out all other possible granulomatous disorders, a sarcoidosis-like reaction was diagnosed based on the identification of characteristic non-caseating granulomas with giant cells in a biopsy taken from the tongue ulcer. The signs and symptoms the patient had experienced resolved following the discontinuation of adalimumab, along with the administration of steroids and methotrexate [73].

## Conclusions

Oral granulomatous disorders can arise secondary to several etiologies—including reactions to foreign bodies, infection, immune dysregulation, proliferative disorders, medications, illicit drugs, and hereditary diseases. Oral lesions vary in presentation and may present as non-specific mucosal swellings, nodules, bone necrosis, or ulceration.

Several diagnostic investigations are suggestive of a granulomatous disorder, including immunohistochemical stains of microorganisms (TB, syphilis, fungi), as well as serological analysis of ACE, ANCA, and ASCA levels to investigate for sarcoidosis, GPA and CD, respectively. Additionally, endoscopy is recommended to rule out CD, and a chest x-ray can display unilateral or bilateral hilar lymphadenopathy that is suggestive of TB and sarcoidosis, respectively.

Obtaining a biopsy is key for the diagnosis of a granulomatous disorder. Some histopathological features can also be helpful, such as the demonstration of caseating necrosis, which is often correlated with infectious disorders (TB and deep fungal infection) while non-caseating necrosis is often found in inflammatory conditions (sarcoidosis and CD). Nevertheless, correlation with clinical microbiology, such as fungal microbiology, may be indicated for the definitive diagnosis. Treatments of oral lesions involve the removal of local or systemic causes, or the administration of topical or systemic therapies.

## Data Availability

No datasets were generated or analysed during the current study.
